# Editorial: Nanoparticles: phytostimulatory and/or phytotoxic influences

**DOI:** 10.3389/fpls.2023.1287686

**Published:** 2023-10-04

**Authors:** Mostafa K. Sarmast, S. Dutta Gupta, Jie Zhou

**Affiliations:** ^1^ Department of Horticultural Science and Landscape Engineering, Faculty of Plant Production, Gorgan University of Agricultural Sciences and Natural Resources (GUASNR), Gorgan, Golestan, Iran; ^2^ Agricultural and Food Engineering Department, Indian Institute of Technology, Kharagpur, India; ^3^ Department of Horticulture, Zhejiang University, Hangzhou, China

**Keywords:** nanomaterial, plant nanobiotechnology, gene expression, plant growth stimulation, nanofertilizers

Nanotechnology is one of the fastest growing fields in science that deals with matters at the nanoscale level. In particular, various fields of biological science have been advanced with spectacular achievements of nano-science and technology. The domain of plant science is of no exception. Over the past years, the nutritional and stimulatory roles of different forms of nanoparticles (NPs) on plant growth and development have been demonstrated. However, the application of NPs in enhancing general plant growth appears to be inconsistent and varies with plant species (Kim et al., 2017; Shang et al., 2019). This clearly highlights that there is an essential need to explore the complexities involved in NP modes of action in suppressing or stimulating plant growth. The nutritional stimulating effect of some metal-NPs and their toxicity effect have been revealed at different NP concentrations. Nanoparticles have an impact on many aspects of plant growth and development mostly through plant hormones, nutritional roles, and control of microbial content of plant and soil (Verma et al., 2022). This Research Topic reveals new aspects of some plant responses to metallic and non-metallic NP. Some of these NPs influence plants by their nutritional effect, and other NPs have been able to affect plant growth via complicated pathways because these NPs have specific physicochemical properties. Nanofertilizers (NFs) usually ameliorate the loss in nutrients from soil and cause greater nutrient use efficiency (NUE) with smart delivery system (Verma et al., 2022). They can significantly increase crop production and do less harm to the environment, compared to the unfavorable use of conventional fertilizers (Verma et al., 2022). Certainly, non-lethal concentrations of metal NPs have interesting stimulatory effects at different stages of plant development. NPs play a regulatory role in the general stress response via a wide range of actions, such as tuning cellular metabolites, hormonal activities, and gene expression (Sarmast and Salehi, 2016). The interaction between some NPs with soil microorganisms and even plants can be significant. The role of metal-NPs in integrated defense mechanisms towards abiotic stress tolerance *in vitro* has also been demonstrated (Jalil and Ansari, 2019). The findings presented by several groups of researchers clearly revealed the potential of plant nanobiotechnology and its outstanding prospect towards plant improvement. A comprehensive approach towards the elucidation of beneficial and adverse effects of metal NPs on plant growth and development is yet to be determined.

Mosa et al. carried out research on *Citrus limon* L. cv. ‘Eureka’ trees after applying silver NPs (AgNPs) at different concentrations. These researchers reported that AgNPs improved the plant yield, vegetative growth, and fruit quality. Since the AgNPs were applied by foliar spraying, the treatments substantially increased vegetative vigor, flower-set and fruiting percentages, tree yield (kg.tree^-1^.ha^-1^), compared to non-treated trees in both years of the experiment. The AgNPs were more effective at higher concentration (10 mg/L).

The AgNP-treated fruit peels and leaves of ten-year-old-lemon were hydrodistillated to extract essential oils (EOs). The GC-MS analysis of extracts revealed the existence of several components, such as geranial, neral, D-limonene, neryl acetate, geraniol acetate, citronellal, and linalool. According to the findings, the EOs of lemon peel had a more potent effect against *Sitophilus oryzae* (a pest) in comparison to leaf-derived EOs based on contact and residual application methods. However, the EOs from the peel were less effective than that from the leaf regarding antifungal activity against *Fusarium solani*.

Sohail et al. explored how *Brassica napus* L. varieties Faisal canola and Shiralee respond to zinc NP (ZnNPs) at a molecular level. ZnNPs were synthesized biologically via aqueous extracts of *Mentha arvensis*, and the researchers described the NP through measurements involving scanning electron microscopy, transmission electron microscopy, X-ray diffraction, and UV-visible spectrophotometry. One-week old canola plants were foliar sprayed with biosynthesized ZnNPs at different concentrations. The molecular effects of ZnNPs on plant growth and development were mostly studied through qRT-PCR and mass spectrometry. Fifteen mg/L of ZnNPs greatly influenced growth vigor, chlorophyll content, and carotenoid concentration in *Brassica napus* L. seedlings. The researchers concluded that an increase in biomass accumulation, relative-water status, carotenoid concentration, chlorophyll content contributed to alterations in gene expression concerning photosynthetic pathways, superoxide dismutase activity, response to biotic stresses, defense mechanisms, and ribosomal compartments in the cellular structure, as revealed by proteomic and transcriptomic analysis.

Verma et al. reviewed the different benefits of versatile NFs and carbon-based nanomaterials for crop improvement in agriculture. Researchers reviewed the role of NFs in soils and plants. They described the phytostimulatorty and phytotoxicity effects of different NFs on different plants subjected to drought, salinity, water logging/flooding, high temperature, freezing stress, UV radiation, nutrient imbalance, and heavy metal toxicity. Discussions addressed how NF application in soil-based irrigation would be followed by NF mobility via symplastic and apoplastic pathways upon NF entry into plant cells. The toxicity and non-toxicity of different NFs on the soil microbial population were also discussed.

So far, there has been an insignificant amount of knowledge on how nanocarriers facilitate and affect nitrogen (N)-based fertilizer mobility, especially regarding their molecular aspects and physiological roles when carried into plant cells. Thus, Feil et al. used urea-doped amorphous calcium-phosphate (U-ACP) NPs as a nanofertilizer for *Cucumis sativus* L. (a model plant). The aim was to fathom how plant cells respond physiologically and stage molecular changes due to the cellular uptake of urea. The researchers described the U-ACP via X-ray powder diffraction, Fourier-transformed infrared spectroscopy, elemental analysis and inductively coupled plasma optical emission spectrometry analyses. The cucumber plants were evaluated for urea uptake and cellular response. Then, 7-day-old starved seedlings were exposed to 1 mM CO(NH2)2 (urea 1 mM) as full-strength U-ACP. Also, half-strength U-ACP treated and control plants were considered for comparison. At different time intervals, root and shoot samples were collected to determine urea uptake by analyzing the total N amount, seedling biomass, root morphological features, leaf relative chlorophyll content (measured by portable Minolta SPAD-502), ionomic profile, and *Cucumis sativus* L. urea active transporter gene (*CsDUR3)* expression. The researchers indicated that the NPs release urea gradually, thereby contributing to plant growth by up-regulating urea uptake dynamics in broader time spans, compared to using bulk-urea as a conventional application on plants. The particular status of NPs caused a threefold increase in the N content of shoots, when comparing it to that in the control group. Furthermore, the U-ACP improved phosphate and calcium concentrations in cucumber tissues, thereby benefiting crop yield, plant growth, and the nutritional merits of the produce. Considering the expansion of the application of nanoparticles in many fields related to agriculture, such as their regulatory role in biotic and abiotic stress, NP-based nutrients as a suitable substitute for traditional fertilizer, the antimicrobial activity of NPs and their interaction with plant cell and soil microorganisms, more experiments are recommended to improve current knowledge. It is necessary to continue to explore the many unknown aspects of these nanoparticles with the help of new technologies.

## Author contributions

MS: Conceptualization, Supervision, Validation, Writing – original draft. SG: Writing – review & editing. JZ: Writing – review & editing.
